# Proposal of serovars 17 and 18 of *Actinobacillus pleuropneumoniae* based on serological and genotypic analysis

**DOI:** 10.1016/j.vetmic.2018.02.019

**Published:** 2018-04

**Authors:** Janine T. Bossé, Yanwen Li, Rita Sárközi, László Fodor, Sonia Lacouture, Marcelo Gottschalk, Maria Casas Amoribieta, Øystein Angen, Katerina Nedbalcova, Matthew T.G. Holden, Duncan J. Maskell, Alexander W. Tucker, Brendan W. Wren, Andrew N. Rycroft, Paul R. Langford, on behalf of the BRaDP1T consortium

**Affiliations:** a Section of Paediatrics, Department of Medicine, Imperial College London, St. Mary's Campus, London, UK; b Department of Microbiology and Infectious Diseases, University of Veterinary Medicine, Budapest, Hungary; c Groupe de Recherche sur les Maladies Infectieuses du Porc, Faculté de Médecine Vétérinaire, Université de Montréal, Québec, Canada; d OVISLAB S.L., Barcelona, Spain; e Department of Microbiology and Infection Control, Statens Serum Institut, Copenhagen, Denmark; f Veterinary Research Institute, Hudcova 70, 621 00 Brno, Czech Republic; g The Wellcome Trust Sanger Institute, Hinxton, UK; h Department of Veterinary Medicine, University of Cambridge, Cambridge, UK; i Faculty of Infectious & Tropical Diseases, London School of Hygiene & Tropical Medicine, London, UK; j Department of Pathology and Pathogen Biology, The Royal Veterinary College, Hawkshead Campus, UK

**Keywords:** *Actinobacillus pleuropneumoniae*, Serovar 17, Serovar 18, Capsule genes, Diagnostics, PCR

## Abstract

The aim of this study was to investigate isolates of *Actinobacillus pleuropneumoniae* previously designated serologically either as non-typable (NT) or as ‘K2:07’, which did not produce serovar-specific amplicons in PCR assays.

We used whole genome sequencing to identify the capsule (CPS) loci of six previously designated biovar 1 NT and two biovar 1 ‘K2:O7’ isolates of *A. pleuropneumoniae* from Denmark, as well as a recent biovar 2 NT isolate from Canada. All of the NT isolates have the same six-gene type I CPS locus, sharing common *cpsABC* genes with serovars 2, 3, 6, 7, 8, 9, 11 and 13. The two ‘K2:O7’ isolates contain a unique three-gene type II CPS locus, having a *cpsA* gene similar to that of serovars 1, 4, 12, 14 and 15. The previously NT isolates share the same O-antigen genes, found between *erpA* and *rpsU*, as serovars 3, 6, 8, and 15. Whereas the ‘K2:O7’ isolates, have the same O-antigen genes as serovar 7, which likely contributed to their previous mis-identification. All of the NT and ‘K2:O7’ isolates have only the genes required for production of ApxII (*apxIICA* structural genes, and *apxIBD* export genes).

Rabbit polyclonal antisera raised against representative isolates with these new CPS loci demonstrated distinct reactivity compared to the 16 known serovars. The serological and genomic results indicate that the isolates constitute new serovars 17 (previously NT) and 18 (previously ‘K2:O7’). Primers designed for amplification of specific serovar 17 and 18 sequences for molecular diagnostics will facilitate epidemiological tracking of these two new serovars of *A. pleuropneumoniae.*

## Highlights

• Identification of two new serovars of *Actinobacillus pleuropneumoniae.*
• Serological confirmation of specific reactivity with homologous antisera.
• Characterization of the capsule loci of serovars 17 and 18.
• Development of PCRs for molecular diagnostics.

## 1 Introduction

*Actinobacillus pleuropneumoniae* is a member of the *Pasteurellaceae* family, and an important respiratory pathogen of swine. Economic losses in the swine industry worldwide are due to mortality associated with acute disease, and the medication cost for treatment and reduced production associated with chronic pleuropneumonia (Sassu et al., 2017). Approaches to controlling the disease include good husbandry/biosecurity and vaccination to reduce incidence, and in the face of clinical disease, treatment with antibiotics to limit severity and spread (Sassu et al., 2017).

It is useful to classify isolates of *A. pleuropneumoniae* not only for epidemiological purposes, but also to inform vaccine development (Gottschalk, 2012). Broadly, isolates can be separated into two biovars, with the more prevalent biovar 1 type requiring exogenous nicotinamide adenine dinucleotide (NAD) for growth, and biovar 2 being NAD-independent (Pohl et al., 1983; Maldonado et al., 2009). Isolates can be further differentiated into serovars, based mainly on capsule (CPS) antigens (Perry et al., 1990; Dubreuil et al., 2000), and 16 serovars are currently recognized (Sárközi et al., 2015; Bossé et al., 2017a).

With improved molecular diagnostics, including whole genome sequencing, it is possible to re-evaluate previously non-typable (NT) isolates, as well as those which present unusual serological or molecular diagnostic results. Recently, Morioka et al. (2016) showed that the methods of antigen preparation and serological testing, could influence the results for identification of certain serovars. Specifically, autoclaved antigens of forty-seven isolates proved un-typable in the agar gel diffusion assay, whereas rapid slide agglutination using formalin-fixed antigens identified all but two isolates as serovars 1, 2 or 15. The CPS types were confirmed by multiplex PCRs (mPCRs) (Bossé et al., 2014; Turni et al., 2014; Ito, 2010), with the two remaining serologically NT isolates producing specific amplicons for serovars 2 and 15. Not all isolates of a given serovar were identified by all of the available mPCRs. The reason for the discrepancy between molecular and serological typing for these two isolates was not investigated; however it is possible that the presence of a transposon insertion in one of the biosynthetic or export genes resulted in no serologically detectable CPS being produced. This was recently shown by Ito et al. (2016) who found that the presence of IS*Apl1* insertions in the CPS loci of two isolates with serovar 15-specific capsule genes rendered them serologically NT.

The aim of this study was to investigate isolates of *A. pleuropneumoniae* previously designated serologically either as NT or as ‘K2:07’, but which did not amplify serovar-specific amplicons in mPCRs (Jessing et al., 2003; Schuchert et al., 2004; Angen et al., 2008), by whole genome sequencing in order to determine the molecular organization of their CPS biosynthetic loci. Our results demonstrate the existence of two new serovars, 17 and 18, of *A. pleuropneumoniae*.

## 2 Materials and methods

### 2.1 Clinical *A. pleuropneumoniae* isolates used in this study

Information regarding the proposed serovar 17 and 18 *A. pleuropneumoniae* isolates sequenced in this study is shown in Table 1. The Danish biovar 1 isolates previously designated as serologically NT were shown to belong to a distinct phylogenetic cluster (*c*17) by amplified fragment length polymorphism (AFLP), and were all recovered from pigs on the same farm (Kokotovic and Angen, 2007). Those designated as ‘K2:O7’ (also biovar 1) had been typed serologically by latex agglutination, but showed no serovar-specific bands when tested by mPCR (Angen et al., 2008; Jessing et al., 2003; Schuchert et al., 2004). The Canadian biovar 2 isolate, 14-022, recovered from the pneumonic lung of a pig in Ontario in 2014, was also found to be NT by serological and molecular methods (Bossé et al., 2014). Information regarding nine additional isolates typed as serovar 17 or 18 by PCR is shown in Table 2. Of these, two of the ‘K2:O7’ isolates (15360/96 and 12323/98) were previously reported to belong to the distinct AFLP phylogenetic cluster, *c*11 (Kokotovic and Angen, 2007).

**Table 1:** Proposed serovar 17 and 18 isolates of *A. pleuropneumoniae* sequenced in this study.

Strain ID	Year	Country	Previous Designation	Biovar	Proposed Serovar	Reference
16287-1	1997	Denmark	NT	1	17	(Kokotovic and Angen, 2007)
17102-11	1997	Denmark	NT	1	17	(Kokotovic and Angen, 2007)
10907-11	1998	Denmark	NT	1	17	(Kokotovic and Angen, 2007)
11990-6	1998	Denmark	NT	1	17	(Kokotovic and Angen, 2007)
11990-7	1998	Denmark	NT	1	17	(Kokotovic and Angen, 2007)
17102-10	1997	Denmark	NT	1	17	(Kokotovic and Angen, 2007)
14-022	2014	Canada	NT	2	17	This study
7311555	2001	Denmark	K2:O7	1	18	This study
7603757	2005	Denmark	K2:O7	1	18	This study

**Table 2:** Additional *A. pleuropneumoniae* serovar 17 and 18 isolates identified by PCR.

Strain ID	Year	Country	Previous Designation	Biovar	Serovar by PCR	Reference
11-008-1	2008	Germany	NT	1	18	This study
1055935	2011	Italy	NT	1	18	This study
A05-0208	2016	USA	NT	2	17	This study
16-006	2016	USA	NT	2	17	This study
17-027	2017	USA	NT	2	17	This study
APP199	2004	Spain	NT	1	18	This study
4436	2017	Spain	NT	1	18	This study
4443	2017	Spain	8	1	17	This study
4461	2018	Spain	8	1	17	This study
15360/96	1996	Denmark	K2:O7	1	18	(Kokotovic and Angen, 2007)
12323/98	1998	Denmark	K2:O7	1	18	(Kokotovic and Angen, 2007)
10374/02	2002	Denmark	K2:O7	1	18	(Kokotovic and Angen, 2007)

### 2.2 Genome sequencing and analysis

Genomic DNA was prepared from all isolates as previously described (Bossé et al., 2015). The Microbes NG Sequencing Facility (www.microbesng.uk) generated and assembled the whole genome sequence of isolate 14-022; for all other samples, paired-end sequencing (Illumina HiSeq 2000) was performed at the Wellcome Trust Sanger Institute (Cambridge, UK), analysed, and assembled as previously described (Bossé et al., 2017b; Howell et al., 2013; Weinert et al., 2015).

Genes of the CPS biosynthetic loci were identified in the draft genomes of each isolate by searching, using BLASTn (http://blast.ncbi.nlm.nih.gov/Blast.cgi), for the *cpxD* gene (accession AIA09380) from the capsule export locus, common to all serovars. The identified loci were analyzed using BLASTn and BLASTx. Multiple sequence alignments were performed using ClustalW in MacVector v15.5.2. Genes encoding Apx toxin biosynthetic and export proteins were also identified by BLASTn using sequences of *apxICABD* and *apxIICA* from the L20 genome (accession number CP000569), and *apxIIICABD* from the JL03 genome (accession number CP000687). The O-antigen biosynthesis loci were identified by tBLASTn using the *erpA* and *rpsU* genes (accession numbers WP_005617946 and WP_005598703, respectively) to delineate the flanking genes.

The sequences of the complete capsule loci for the serovar 17 and 18 isolates listed in Table 1 have been deposited in GenBank under accession numbers: MG780416 - MG780424.

### 2.3 Production of antisera

Hyperimmune rabbit polyclonal antisera were raised against formalin-killed preparations of isolates 16287-1 (Danish NT; proposed reference strain for serovar 17) and 7311555 (Danish ‘K2:O7’; proposed reference strain for serovar 18), as previously described (Sárközi et al., 2015).

### 2.4 Serological testing

Indirect hemagglutionation (IHA) was performed using antisera raised against the 16 recognised serovars, as well as the new antisera raised in this study, as previously described (Sárközi et al., 2015).

### 2.5 Diagnostic PCRs

**Fig. 1:**
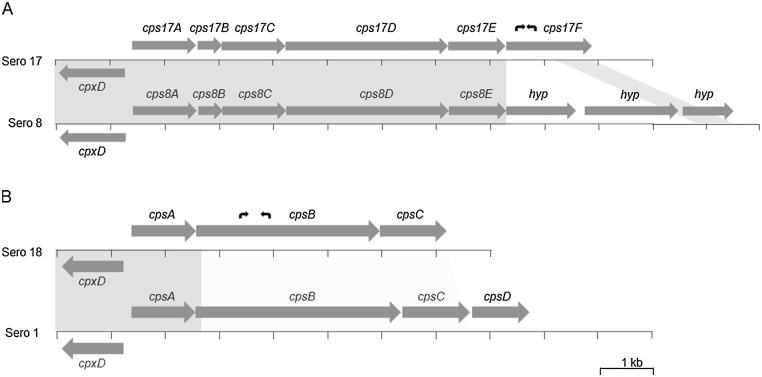
Schematic representations of *A. pleuropneumoniae* CPS biosynthetic loci. A) The serovar 17 type I CPS biosynthetic locus and comparison to that of the serovar 8 reference strain, 405. The CPS biosynthesis genes for serovar 17 (Sero 17; *cps17ABCDEF*). The serovar 8 CPS biosynthetic locus shares the first five genes as the serovar 17 locus (99% identity at the nucleotide level as indicated by the grey box joining these genes), and contains three further genes (each labelled *hyp* to indicate that they encode hypothetical proteins), the last of which shares 91% identity at the nucleotide level with the 3′ end of the *cps17F* gene (as indicated by the lighter grey parallelogram). B) The serovar 18 type II CPS biosynthetic locus (*cps18ABC*) and comparison to that of the serovar 1 reference strain, 4074 (*cps1ABCD*). The *cpsA* genes in these serovars share 99% identity at the nucleotide level (indicated by the grey box joining the genes), whereas the *cpsBC* genes share less than 55% identity at the nucleotide level (indicated by the pale grey trapezoid joining these genes). The locations of serovar 17- and 18-specific primers are indicated as small curved black arrows above *cps17F* and *cps18B*, respectively. The relative size and location of each gene are indicated by the sizes and positions of arrows (solid dark gray). In all loci shown here, the CPS biosynthesis genes are located downstream of, and on the opposite strand to, the capsule export genes common to the loci in all *A. pleuropneumoniae* serovars (*cpxDCBA*; only *cpxD* is shown).

Primers AP17F (TTGTAATGGCGGTGTAATGCTAC) and AP17R (CATAAGTGCAGCCATCTCTTTCAG) were designed to amplify a 302 bp serovar 17-specific amplicon; and primers AP18F (CGGAGTTTGGCAGCATAAAGG) and AP18R (CCATAATCGGTGCTCAACTAAGAATG) were designed to amplify a 514 bp fragment of the serovar 18-specific amplicon (see Fig. 1 for locations of primers). These primers were combined, along with those previously designed (Bossé et al., 2014) for detection of the 418 bp *apxIV* band common to all serovars, and were initially tested using genomic DNA from the one Canadian and six Danish NT isolates (for serovar 17), and the two Danish ‘K2:O7’ isolates (for serovar 18), shown in Table 1, as well as the reference strains for the 16 known serovars strains (i.e. 4074^T^, 1536, S1421, M62, K17, L20, Femø, WF83, 405, CVJ13261, D13039, 56153, 8328, N-273, 3906, HS143, and A-85/14, respectively). Subsequently, the specificity of the primers was further evaluated using the same 68 clinical isolates of *A. pleuropneumoniae* (covering serovars 1–15), the six *A. pleuropneumoniae* serovar 16 isolates, and the 31 porcine-associated species recently used for evaluation of serovar 16-specific primers (Bossé et al., 2017a), as well as the nine clinical isolates shown in Table 2.

## 3 Results and discussion

The serovar 17 isolates (Table 1; proposed reference strain 16287-1) encode a type I CPS locus (Fig. 1A), with the first three genes (*cps17ABC*) encoding a glycerol transferase, a glycerol-3-phosphate cytidylyltransferase, and a hypothetical protein, respectively, with high identity to those encoded by the first three genes in the loci of serovars 2, 3, 6, 7, 8, 9, 11 and 13 (Xu et al., 2010; Bossé et al., 2014). The serovar 17 CPS locus most closely resembles that of serovar 8, with 99% identity at the nucleotide level between the sequences *cps17ABCDE* and *cps8ABCDE* (Fig. 1A). The *cpsDE* genes in both these serovars encode the same predicted glycosyltransferases. We previously reported (Bossé et al., 2014) that the CPS locus of the serovar 8 reference strain, 405, was comprised of these five genes. However, upon further analysis of the sequences downstream of the *cpsE* gene in the draft genome sequence of strain 405, and in the complete genome sequence of MIDG2331 (Bossé et al., 2016), a further three ORFs encoding hypothetical proteins (MIDG2331_01764, MIDG2331_01763, and MIDG2331_01762) are present that may also be part of the CPS biosynthetic locus, though the function of these proteins has yet to be determined.

In the serovar 17 CPS locus, the first five genes are followed by a single ORF (*cps17F*) predicted to encode a hypothetical protein containing a DUF1919 superfamily domain (involved in cell wall/membrane/envelope biogenesis) in the N-terminal portion of the sequence. The first 197 amino acids (AAs) of the Cps17F sequence shares 54% identity with a predicted expolysaccharide biosynthesis protein (206 AA) encoded by a gene divergently transcribed from that encoding phosphomanomutase in all sequenced serovars of *A. pleuropneumoniae* (see accession number WP_005603976), including the draft genomes of the serovar 17 and 18 isolates from this study. The C-terminal portion of the Cps17F protein (AAs 229–515) shares 94% identity with the 303 AA protein encoded by the final gene in the serovar 8 MIDG2331 CPS locus, MIDG2331_01762 (Fig. 1), suggesting that the serovar 17 CPS locus may have arisen by recombination of a gene encoding a predicted expolysaccharide biosynthesis protein into the terminal gene of the serovar 8 CPS locus, with deletion of the preceding two serovar 8 CPS locus genes.

The serovar 18 isolates (Table 1; proposed reference strain 7311555) encode a type II CPS locus (Fig. 1B), with the first gene encoding a phosphotransferase sharing high identity with that encoded by the first gene in the loci of serovars 1, 4, 12, 14, and to a lesser extent with that of serovar 15 (Xu et al., 2010; Ito, 2015; Ito and Sueyoshi, 2015). The second gene in the serovar 18 locus encodes a 1115 AA glycosyltransferase with only 32% identity to the 1247 AA glycosyltransferase encoded by the serovar 1 *cpsB* gene, although the first 23 AAs are identical in the two proteins. The final gene in the serovar 18 CPS locus encodes a 401 AA protein similar to the 406 AA protein encoded by the *cps1C* gene, though they share only 41% identity. These proteins contain an Asp2 superfamily domain found in Accessory Sec system GspB-transporters of Gram positive bacteria. The serovar 18 CspC protein shares highest identity (55% over 361 AAs) with a predicted alpha/beta hydrolase from *Cronobacter malonaticus* (accession number WP_032971501), whereas the serovar 1 CpsC shares highest identity (68% over 387 AAs) with a predicted Accessory Sec system GspB-transporter from *Basfia succiniciproducens* (accession number SEP68412). The function of these CpsC proteins has yet to be determined. In the serovar 1 CPS locus, there is an additional gene, *cpsD*, encoding a 347 AA predicted acetyltransferase that is not present in the serovar 18 locus.

Results of IHA using hyperimmune rabbit sera revealed homologous reactivity (titer of 2560) of the serovar 17 antiserum with the proposed reference strain (16287-1), with similar titers (+/- one dilution) for other serovar 17 isolates, including 14-022, confirming both biovar 1 and biovar 2 isolates of serovar 17. Cross-reactivity (i.e. titers ≥640) was also detected between serovars 17 and 8 (titer of 1280 for the serovar 17 serum tested with the serovar 8 strain; but only a titer of 640 for the serovar 8 serum tested with the serovar 17 strain). In contrast, the serovar 18 antiserum was highly specific, giving a titer of 10,240 with the proposed reference strain 7311555. These results are not surprising given the genetic data showing more unique genes for the serovar 18 locus, and the shared genes common to the CPS loci of serovars 8 and 17.

We identified the O-antigen genes in the sequenced serovar 17 and 18 isolates, located between the *erpA* and *rpsU* genes, as previously reported for other serovars of *A. pleurpneumoniae* (Xu et al., 2010). BLASTn results for the serovar 17 O-antigen genes indicated 97–100% identity (over 13,600 bp) with the loci in serovars 3, 6, 8, 15 (accession numbers CP000687, ADOG00000000, LN908249, and AB743837, respectively). Given that we only found serological cross-reactivity between serovar 17 and the serovar 8, it would appear that our IHA assay detected antibodies directed against CPS antigens, but not O-antigens. BLASTn analysis of the serovar 18 O-antigen genes revealed 97% identity (over 11,340 bp) with the locus in serovar 7 (accession number CP001091). Again, our IHA results did not show cross-reactivity between serovars 18 and 7, indicating that antibodies directed against common O-antigens were not detected. However, it is likely that the previous serological designation of ‘K2:O7’ for these isolates was due to detection of O-antigen-specific antibodies.

**Fig. 2:**
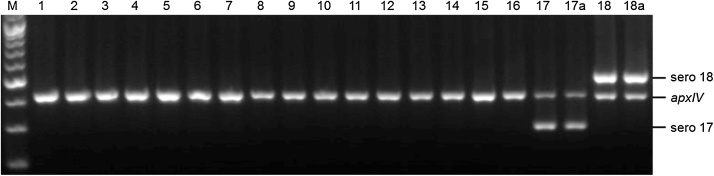
Verification of specificity of primers for molecular identification of *A. pleuropneumoniae* serovars 17 and 18. An *apxIV* (418 bp) amplicon is detected in all 18 serovar reference strains; the serovar 17-specific amplicon (302 bp) is detected only in the serovar 17 isolates (lanes 17, 17a); and the serovar 18-specific amplicon (514 bp) is detected only in the serovar 18 isolates (lanes 18, 18a). Lane M contains molecular size markers (100 bp ladder). Lanes 1 to 18 contain samples from the following strains: 1, 4074T; 2, S1536; 3, S1421; 4, M62; 5, L20; 6, FemØ; 7, WF83; 8, 405; 9, CVJ13261; 10, D13039; 11, 56153; 12, 8329; 13, N-273; 14, 3906; 15, HS143; 16, A-85/14; 17, 16287-1; 18, 7311555. Lane 17a contains the biovar 2 isolate, 14-022, showing the same serovar 17 amplicon as the biovar 1 reference strain and other biovar 1 clinical isolates tested. Lane 18a contains serovar 18 isolate 7603757.

For molecular diagnostics, we designed primers to amplify a unique 302 bp amplicon from the serovar 17 *cpsF* gene, and a unique 514 bp amplicon from the serovar 18 *cpsB* gene. When multiplexed, along with previously designed species-specific *apxIV* primers for detection of all *A. pleuropneumoniae* serovars (Bossé et al., 2014), specific detection of the serovar 17 and 18 amplicons was seen with the respective proposed reference strains, with all of the other serovar reference strains only amplifying the *apxIV* amplicon (Fig. 2). The specificity of the serovar 17 and 18 primers was further confirmed using the other sequenced serovar 17 and 18 isolates shown in Table 1, as well as DNA from clinical isolates of *A. pleuropneumoniae* representing the 16 known serovars, other bacteria associated with pigs, and by virtual PCR using genome sequences available in Genbank, as previously described (Bossé et al., 2017a).

PCR analysis of DNA samples from the isolates shown in Table 2 revealed that the recent biovar 2 NT isolates from the USA typed as serovar 17, as did two of the recent biovar 1 Spanish isolates (4443 and 4461). These two Spanish isolates were initially typed as serovar 8 by mPCR (Bossé et al., 2014), whereas all of the North American biovar 2 isolates were NT in this assay. These results indicate that some serovar 17 isolates share the *cpsAB* sequences targeted by what were thought to be serovar 8-specific primers. Thus, in the past, some serovar 17 isolates may have been molecularly typed as serovar 8, depending on the primers used, or serologically as 3/6/8 if the test used detected O-antigens. Evidence supporting serovar 17 as separate from serovar 8 includes the segregation of the Danish NT isolates by AFLP into the *c*17 cluster distinct from serovar 8 isolates in *c*5 (Kokotovic and Angen, 2007); the lack of *apxIIIABCD* genes normally found in serovar 8 (see below); and the presence of only six CPS biosynthetic genes compared to eight genes found in the CPS locus of serovar 8.

The previously NT isolates from Germany (2008), Italy (2011), and the two Spanish NT isolates (APP199 and 4436; from 2004 and 2017, respectively), typed as serovar 18, as did three additional’ K2:O7’ isolates collected between 1996–2002. These’ K2:O7’ isolates included 15360/96 and 12323/98, that had been shown to form a distinct AFLP cluster (*c*11), separate from other K2:O7 isolates found in cluster *c*8 (Kokotovic and Angen, 2007). Whereas the K2:O7 isolates in *c*8 were found to produce amplicons for serovar 2, as well as serovar 8, when tested in mPCRs (Jessing et al., 2003; Schuchert et al., 2004), those in *c*11 only produced a species-specific *omlA* amplicon (Jessing et al., 2003). The primer pair used by Schuchert et al. (2004) for detection of serovar 8 was designed to amplify a 977 bp sequence from the *cpsAB* genes which are common to serovars with type I CPS loci, with different degrees of identity at the nucleotide level; whereas the serovar 2 primer pair amplified region spanning the *cpsCD* genes, the latter being less well conserved amongst type I CPS loci. Likewise, the serovar 2 primer pairs used by Jessing et al. (2003) were designed to amplify a 504 bp *cpsD*-specific region. Thus, the serovar 2 amplicon produced by the K2:O7 isolates in AFLP cluster *c8* is likely specific for the serovar 2 CPS locus, whereas the serovar 8 amplicon is likely due to the presence of common priming sites in the *cpsAB* genes.

The distribution of genes encoding Apx toxins I-III differ depending on serovar, with some biovar 2 serovars showing different Apx toxin profiles than their biovar 1 counterparts (Sthitmatee et al., 2003; Rayamajhi et al., 2005; Maldonado et al., 2009). Toxin typing PCRs have been used for characterizing isolates, since those producing ApxI tend to be more highly virulent (Frey et al., 1995; Sthitmatee et al., 2003; Rayamajhi et al., 2005; Maldonado et al., 2011). The serovar 17 and 18 isolates sequenced in the current study, regardless of biovar, were all found to harbor only the genes required for production of ApxII (*apxIICA* structural genes, and *apxIBD* export genes), a pattern similar to that reported for biovar 1 serovars 7 and 12, as well as biovar 2 isolates of serovars 2, 4, 7, 11 and 13 (Frey et al., 1995; Rayamajhi et al., 2005; Maldonado et al., 2009, 2011). A number of Spanish biovar 2 NT isolates were also reported to produce only ApxII (Maldonado et al., 2009, 2011). Testing of these isolates with our new mPCR would determine if any belong to serovars 17 or 18.

In conclusion, we have used a combination of whole genome sequencing and serology to clarify the status of previously NT isolates, as well as those previously mis-typed as ‘K2:O7’ (and unreactive in previous CPS-specific mPCRs), showing that they represent new serovars, 17 and 18 respectively, of *A. pleuropneumoniae*. Biovar 1 isolates of serovar 17, first recovered in Denmark in 1997, appear to still be in circulation in Europe, with a recent (2017) isolate from Spain identified. Whereas biovar 2 serovar 17 isolates have been recovered in North America since 2014. Given the relatedness of the core *cps* and O-antigen genes between serovars 8 and 17, it is possible that other isolates of serovar 17 may have been typed as serovar 8 in the past. Serovar 18 isolates (biovar 1) have been in circulation in Europe since 1996, most recently recovered in Spain in 2018. The PCR primers designed in this study will help in further identification and epidemiological tracking of these two newly designated serovars of *A. pleuropneumoniae.*

## Conflict of Interest

We declare that we have no conflict of interest.

## Funding

This work was supported by a Longer and Larger (LoLa) grant from the Biotechnology and Biological Sciences Research Council (BBSRC grant numbers BB/G020744/1, BB/G019177/1, BB/G019274/1 and BB/G018553/1), the UK Department for Environment, Food and Rural Affairs, and Zoetis (formerly Pfizer Animal Health) awarded to the Bacterial Respiratory Diseases of Pigs-1 Technology (BRaDP1T) consortium. MTGH was supported by the Wellcome Trust (grant number 098051). RS and LF were supported by the Hungarian Scientific Research Fund (OTKA 112826). Genome sequencing was provided by MicrobesNG (www.microbesng.uk), which is supported by the BBSRC (grant number BB/L024209/1).
